# The Effectiveness of Teaching the Teacher Interventions in Improving the Physical Activity among Adolescents in Schools: A Scoping Review

**DOI:** 10.3390/healthcare12020151

**Published:** 2024-01-09

**Authors:** Antonios Christodoulakis, Izolde Bouloukaki, Antonia Aravantinou-Karlatou, Katerina Margetaki, Michail Zografakis-Sfakianakis, Ioanna Tsiligianni

**Affiliations:** 1 Department of Social Medicine, School of Medicine, University of Crete, 71003 Heraklion, Greece; izolthi@gmail.com (I.B.); toniakoinerg2@gmail.com (A.A.-K.); katmargetaki@hotmail.com (K.M.); i.tsiligianni@uoc.gr (I.T.); 2 Department of Nursing, School of Health Sciences, Hellenic Mediterranean University, 71410 Heraklion, Greece; mzografakis@hmu.gr

**Keywords:** teaching the teachers, interventions, students, adolescents, physical activity, physical education, school, health, scoping review

## Abstract

Physical inactivity is a leading risk factor for global mortality as it increases the risk of non-communicable diseases and decreases overall health. Therefore, increasing physical activity (PA) is strongly recommended, particularly in adolescents. The Teaching the Teachers (TTT) approach is a prominent example of an intervention that could enhance PA levels in adolescents. However, available evidence on the efficacy of TTT interventions in promoting PA among adolescents is either limited or inconclusive. Therefore, a scoping review was conducted to provide an overview of the current state of knowledge regarding the effectiveness of TTT interventions in improving the PA of adolescent students. The PRISMA-Scoping methodology was employed, and articles in the Medline database were searched. We screened 2357 articles for inclusion, and finally included 16 articles. Most articles were conducted in European countries. The TTT interventions appeared to positively affect various aspects of PA. These aspects include support for personal autonomy, intent to engage in PA, improvements in body composition and fitness level, social support, enjoyment of Physical Education (PE), and positive attitudes towards PE. Consequently, policymakers could utilize TTT interventions to improve the physical activity of adolescents, which may reduce the burden of non-communicable diseases and foster healthier societies.

## 1. Introduction

The World Health Organization (WHO) ranks physical inactivity as the fourth leading risk factor for global mortality [1]. Physical inactivity is positively associated with non-communicable diseases (NCDs) and negatively associated with general health [1,2]. A key strategy for improving physical inactivity is to increase awareness and incorporate interventions that promote physical activity [3] Since physical activity can significantly contribute to the preservation of overall health and mental wellness of individuals while mitigating the likelihood of non-communicable diseases (NCDs) later in life, especially in adolescents [2]. To achieve that, it is recommended that adolescents and young adults engage in physical activity for at least sixty (60) min per day [4]. 

Physical activity (PA) for health could be defined as ‘as any bodily movement produced by skeletal muscles that requires energy expenditure’ [5]. Investing in adolescent health and well-being will provide benefits to adolescents today, future adults, and the next generation of children [6]. Although adolescence is generally characterized by good health, various non-communicable diseases (NCDs) that manifest later in life can be partially attributed to modifiable risk behaviors established during this period. These behaviors include smoking, unhealthy eating habits, and low levels of physical activity [7].

Adolescent health has experienced significant global trends in the past few decades. In 2016, the number of overweight or obese children and adolescents aged 5–19 was almost 340 million worldwide [8]. Moreover, there is a growing prevalence of mental health disorders among adolescents, such as depression and anxiety [9]. To minimize the impact of NCDs on individuals and society, a comprehensive approach is required. Such an approach would require the participation of all sectors, including education, to mitigate the risks associated with NCDs and facilitate interventions for their prevention and control [3].

Studies have shown that adolescents do not achieve the recommended sixty (60) min of daily exercise [10,11,12], even at school [13]. Therefore, worldwide, it is crucial to create and implement efficient interventions to encourage and maintain physical activity among adolescents. For this purpose, a variety of interventions have been developed and evaluated to improve PA in adolescents, such as school-based, parent-based, community-based, and technology-based interventions [14,15]. One example of an intervention that could improve the PA of adolescents is the “Teaching the Teachers” (TTT) approach. The TTT approach involves providing training/support to teachers so that they can more effectively achieve the educational objectives of a subject, such as improving the physical activity (PA) of their students [16]. These interventions can be implemented through workshops, coaching, mentoring, peer observation, feedback, etc. [17]. Additionally, TTT interventions have the potential to enhance the professional development of teachers [18]. By participating in these programs, teachers can expand their knowledge, refine their skills, align their attitudes and beliefs, and modify their behaviors, thereby contributing to their overall effectiveness in the classroom [19]. Through the teacher, TTT interventions may also help enhance the students’ academic performance, drive, participation, and overall well-being [19]. Furthermore, the TTT approach presents several potential advantages, such as the ability to reach a substantial and varied group of adolescents, leveraging existing educational resources and infrastructure, improving the rapport between instructors and students, and fostering a supportive academic environment for physical activity [20]. However, evidence on the effectiveness of TTT interventions in improving PA among adolescents is either limited or inconsistent, as different studies have used different methods, outcomes, and criteria to evaluate the interventions [21,22]. Therefore, this scoping review aimed to provide an overview of the current state of knowledge on the effectiveness of TTT interventions in improving PA in adolescent students. 

## 2. Materials and Method

### 2.1. Design

This scoping review was conducted in accordance with the principles recommended in the Preferred Reporting Items for Scoping Reviews (PRISMA-ScR) checklist and explanation [23] and Joanna Brigg’s Institute Reviewer’s Manual for Scoping Reviews [24].

### 2.2. Search Strategy

A comprehensive literature search was performed in one electronic biomedical literature database (MEDLINE) from September to October 2023. We employed the PICO methodology to formulate and refine the research queries for this study (Table 1). Furthermore, we used the following keywords: ‘teaching the teachers’, ‘interventions’, ‘adolescent students’, ‘physical activity’, ‘physical education’, ‘school-based interventions’, ‘health education programs’, ‘health promotion in schools’, ‘teacher training’, and ‘adolescent health’.

### 2.3. Study Inclusion and Exclusion Criteria

Initially, the process of examining titles and abstracts for inclusion was performed by four (4) individual reviewers. After removing duplicates, the reviewers collaborated in two teams (with two reviewers each) and evaluated the remaining studies. In cases of discrepancies between the teams and reviewers, a fifth reviewer was asked to resolve any inconsistencies during the screening process. To facilitate calibration, the reviewers arranged two meetings throughout the process, with the primary meeting centered on establishing a common understanding of the criteria, and the subsequent meeting involved comparing selected studies and discussing any disparities.

Our research included a wide range of studies, including cross-sectional studies, observational studies, interventional trials, longitudinal studies, randomized controlled trials, and qualitative research methods, such as interviews and focus groups, from 2013 to 2023. Studies should have examined the efficacy of interventions designed to teach teachers about the promotion of physical activity among adolescent students. However, our search was limited to studies that were freely available in full text, were conducted in English, and involved adolescents going to school within the age range of 10–18 years. Additionally, these studies should have explored the relationship between physical activity and Teaching-The-Teachers (TTT) interventions and examined the efficiency of the interventions among adolescent students. Conversely, we excluded case reports, case series, commentaries, editorials, letters, conference abstracts, reviews, and book chapters. Additionally, grey literature, including non-peer-reviewed papers (e.g., academic reports and dissertations), was omitted. The selection process involved excluding duplicate publications or multiple reports from the same study, with priority given to the most comprehensive or recent report. 

### 2.4. Procedure 

**Figure 1:**
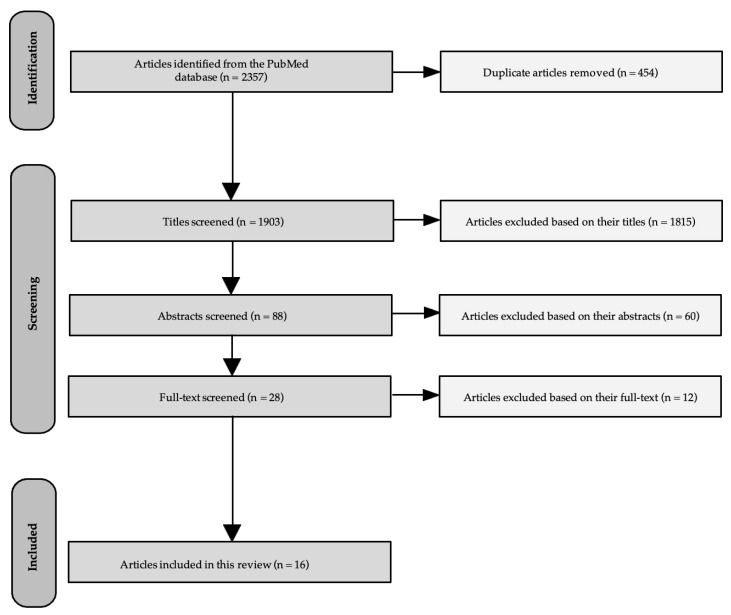
PRISMA flow diagram of the literature search for this review.

The initial database search yielded 2357 articles for this scoping review. After screening and removing 454 duplicates, a total of 1903 articles were screened based on their titles by identifying those that hinted at interventions that involved teachers and improved PA in adolescent populations. Following that, 88 titles met the inclusion criteria and were selected for a second/further evaluation based on their relevance to our aim (TTT interventions to improve PA in adolescent students) and study design as described in their abstracts. Subsequently, 28 abstracts met the inclusion criteria; therefore, their full texts were retrieved for further screening. However, 12 articles were excluded from the 28 retrieved full texts, based on the inclusion/exclusion criteria outlined in the methods section. Thus, 16 full-text articles were finally included in this review. It should be noted that the PRISMA flow diagram for the literature search [25] is presented in Figure 1.

### 2.5. Data Extraction and Analysis

The reviewers utilized a standardized data extraction form to extract information from the full texts, focusing specifically on study design, population characteristics, the TTT intervention, student intervention, outcome measures, and results related to physical activity (PA). Three independent reviewers assessed each form, and any discrepancies were addressed through discussion. The data were compiled from the finalized data extraction form.

## 3. Results

### 3.1. Characteristics of the Included Studies

#### 3.1.1. Study Design and Location

Table 2 provides an overview of the characteristics of the 16 studies included. Of these articles, one (1) was crossover, ten (10) were Randomized Controlled Trials (RCTs), and five (5) were quasi-experimental. In terms of countries in which articles have been published, most studies were carried out in European countries (Spain [26,27,28,29], Finland [30], The Netherlands [31], Germany [32], Estonia [33]), with the remaining contributions coming from Australia [17,34,35], Uruguay [36], Brazil [37], USA [38], and China [39,40]. All articles were published starting from 2013, with fifteen out of the sixteen published starting from 2017. 

#### 3.1.2. Population

The range of schools surveyed in each article spanned from one to forty-six, resulting in a total of two hundred and ten schools. The students were sampled in varying sizes, with participant counts ranging from 55 to 3763. In total, there were 10,311 participants. The study included adolescent students aged between 10 and 16 years. All the articles featured a representative sample encompassing both sexes.

#### 3.1.3. Exposure

The studies implemented various interventions aimed at improving the physical activity of adolescents. In all articles, the participants in the control group followed the established, usual physical education curriculum. Interventions generally involved a form of support and/or guidance, and/or expansion of knowledge to teachers, which was then applied to the established curriculum in an effort to improve at least one aspect related to PA in students. These included the introduction of a biweekly intermittent teaching unit for a duration of eight weeks, accompanied by the implementation of a behavior modification strategy tailored to the specific context [26]; a specific model of a healthy physical education curriculum in the field of physical education for a duration of twelve weeks [40]; a hybrid educational program for eleven (11) weeks that combined the Model of Personal and Social Responsibility (TPSR) with the Teaching Games for Understanding (TGfU) [27]; physical education classes engaged in 15 min cooperative games once per week for seven to fourteen weeks [32]; an enhanced school-based PE intervention program, SPARK PE, a middle school curriculum containing instructional strategies, activities, assessments, and ideas for adapting instruction [38]; classes based on the autonomy support style for eight (8) months [37]; the ¡Activate Ya! Program including after school program, activity breaks, and a final showcase event for one (1) year [36]; a combined strength exercise and motivational program embedded in the school curriculum for one (1) year [31]; a didactic unit planned for the ‘Games and Sport’ content block of the educational system [28]; and expansion of their instruction toolkit for PE [17]. 

#### 3.1.4. Teaching-the-Teacher (TTT) Interventions

The articles highlighted a variety of interventions that focused on instructing educators to promote physical activity among adolescents. One recent study utilized training in autonomy and need support, specifically targeting motivation and behavior change techniques [26]. In this study, teachers received guidelines to facilitate accurate delivery of students’ lessons [26]. Another study included a similar intervention that involved teachers attending training seminars on organizing and conducting classes with an autonomy support style, as well as additional seminars on self-determination theory (SDT) and the Hierarchical Model of Intrinsic and Extrinsic Motivation (HMIEM) [37]. In line with these studies, Sanchez-Oliva et al. established a training program based on SDT to enhance teachers’ interpersonal styles and develop strategies to promote autonomy, competence, relatedness, and need satisfaction [28]. SDT-based interventions were also used by Lonsdale et al., who employed three motivational teaching strategies (explaining relevance, providing choice, and free choice) [35]. Another study employed the theory and practice of a healthy physical education curriculum model to train teachers and attained satisfactory training results [40]. Teachers in a different study had to undergo a sustained training period to facilitate the implementation of other specific instructional courses while receiving feedback, support, and guidance in the application of educational models [27]. Additionally, training allowed teachers to offer their students support in cognitive, organizational, and procedural autonomy while preventing intimidation and negative emotions [33]. In other studies, teachers were trained in cooperative games [32] and in enhanced school-based PE intervention programs, such as SPARK PE [38] and ¡Activate Ya! [36] programs that involved specific strength exercises, along with workshops to improve their motivational speaking skills [31]. Additionally, other studies included a 12 h interactive autonomy support teacher training program with techniques and strategies to promote autonomous motivation towards PA in students [30]; workshops designed to expand their knowledge of Self-Determination Theory and learn how to implement into their lessons the fitness dice and upbeat music [39]; implementation of a Sport Education model based on a hybrid model of Teaching Games for Understanding and Sport Education models [29]; and improvement of their relatedness-supportive teaching practices [34].

#### 3.1.5. Outcomes Measures

Table 2 provides a description of the outcomes measures. The most widely used outcome measures were perceived autonomy support [26,28,30,32,33,34,35,37,40] and motivation towards Physical Education (PE) [17,26,27,28,29,30,33,35,37,40] in nine articles.

Additionally, enjoyment and satisfaction with PE were examined in seven articles [17,27,32,34,37,38,39]. Examining physical activity (PA) levels [26,29,30,31,36,38,40] and satisfaction with basic psychological health [26,27,28,29,33,37] were also widely used outcome measures in six and five articles, respectively. Simultaneously, three articles examined how students perceived their teachers’ behaviors [33,34,35,37], and three articles examined social support [27,32,38]. In addition, the studies also used the following outcome measures: attitudes towards PE classes [38], weekly muscle strengthening [38], body composition assessed by the deuterium dilution technique [31], fitness levels of students, perceived barriers [38], and personal responsibility [27]. All 16 articles used quantitative approaches to quantify each outcome variable. Qualitative approaches were employed in one article [27] as well. The majority of outcomes were evaluated using validated questionnaires (Table 2).

### 3.2. Associations between Teaching-the-Teachers (TTT) Interventions and Outcomes Associated with Physical Activity (PA) in Adolescents

Table 2 provides an overview of the key findings derived from the included 13 articles, which explored the relationship between TTT interventions and various outcomes associated with PA in adolescents. The majority of the reviewed articles (13/16) concurred that TTT interventions demonstrated a positive association with increased levels of physical activity, while the remaining found no association (3/16). Specifically, TTT interventions appeared to positively affect the enhancement of physical activity (mainly PA levels), autonomy support, intention to engage in physical activity (PA), body composition, technical performance, fitness level, social support, enjoyment of Physical Education (PE), and attitudes towards PE classes. However, two articles [17,30] reported no difference between baseline and after post-intervention PA levels.

#### 3.2.1. Physical Activity (PA) Levels

A total of seven articles [17,27,32,34,37,38,39] were identified, with three of them [36,39,40] presenting evidence that supports the positive impact of TTT interventions on improving PA. According to a recent study, TTT interventions were found to increase habitual physical activity levels, as measured by the number of days in the previous week and a typical week, where at least sixty (60) minutes of PA were completed [26]. In support of this, Hao et al. found that a TTT intervention could increase the level of involvement in extracurricular sports activities [40]. Additionally, the implementation of the ¡Activate Ya! intervention resulted in an overall improvement in the past seven-day PA score among the students in the intervention school [36]. In contrast, the SPARK intervention demonstrated varied effects on self-reported physical activity outcomes, indicating that the intervention did not affect daily self-reported physical activity [38]. Moreover, participation in the SPARK intervention demonstrated a negative correlation with adherence to the recommendations for muscle-strengthening exercises [38]. In line with this, Ten Hoor et al. highlighted that all adolescents experienced a decrease in physical activity, regardless of the intervention, after one (1) year [31]. Nevertheless, as a result of the intervention, the intervention group exhibited lower levels of decline in physical activity than the control group [31]. However, an article that tried to improve autonomous motivation for PA did not find any change in PA behavior at post-intervention and at 1 month [30].

#### 3.2.2. Autonomy Support

Teaching-The-Teachers (TTT) interventions seem to improve the perceived autonomy support [26,28,30,32,33,34,35,37,40]. According to Guijarro-Romero et al., the implementation of an intermittent teaching unit resulted in a notable enhancement of students’ cognitive and procedural autonomy support [26]. The introduction of another physical education curriculum model yielded notable improvements in the perceived need for support by students in PE, autonomous motivation in PE, autonomous motivation in leisure time (LT), and promotion of student participation in extracurricular sports [40]. Furthermore, a positive impact was observed in the context of perceived autonomy support when different TTT interventions were used, including a combined intervention group that incorporated both face-to-face and web-based elements [33], a teaching model that promoted autonomy support [37], a training program based on SDT [28], and an intervention that offered choices [35]. In contrast, an intervention program that employed cooperative games did not appear to contribute to a higher sense of autonomy or self-determination among students [32].

#### 3.2.3. Intention and Motivation to Engage in Physical Activity

Teaching-The-Teachers (TTT) interventions, including a healthy physical education curriculum model [40], a hybrid intervention program based on the Teaching Personal and Social Responsibility (TPSR) model and the Teaching Games for Understanding (TGfU) [27], and a training program based on the SDT [28,39], appear to positively influence the intention to participate in physical activity. Other TTT interventions yielded statistically significant improvements in motivation towards physical activity [26,27,33,37]. Conversely, two studies demonstrated that TTT interventions that used intermittent teaching units [26] and provided choice [35] had no influence on motivation towards PE. However, a study based on a hybrid model of teaching games for understanding and sports education did not improve autonomous motivation, relatedness, and intention to engage in PA [29].

#### 3.2.4. Body Composition, Technical Performance, and Fitness Level

Teaching-The-Teachers (TTT) interventions seem to improve body composition, technical performance, and fitness level [31,38]. For example, after incorporating TTT interventions and making minor adjustments to physical education classes, notable changes in body composition were evident, leading to a 1.6% reduction in fat mass in the intervention group compared with the control group [31].

#### 3.2.5. Social Support

Three articles [27,32,38] highlighted the positive effects of TTT intervention on social support. Implementing a hybrid approach that combines the Teaching Personal and Social Responsibility (TPSR) model and the Teaching Games for Understanding (TGfU) model proved to be effective in promoting social and personal responsibility among secondary school students [27]. The use of cooperative games in the intervention program resulted in participants experiencing a greater sense of social connection [32]. Individuals who experienced considerable support from their family and friends were more inclined to have positive attitudes towards PA [38].

#### 3.2.6. Enjoyment of PE and Attitudes towards PE Classes

Some of the included articles [27,29,32,34,37,38,39] identified the positive impact of TTT interventions on perceived enjoyment in physical education classes. For example, interventions based on the self-determination theory that focused on improving the relatedness [34] and understanding regarding students’ requirements for competence, autonomy, and relatedness yielded significantly positive results in the attitudes towards PE classes [39]. Another remarkable example was the positive effect of a hybrid model based on teaching games for understanding and sports education models on students’ enjoyment of PE [29]. However, there was no evidence that the SPARK intervention yielded better PA attitudes [38].

## 4. Discussion

The aim of this scoping review was to provide a comprehensive understanding of the current state of knowledge on the effectiveness of Teaching-The-Teachers (TTT) interventions in enhancing physical activity (PA) among adolescent students. Our main finding was that TTT interventions seem to have a positive effect on physical activity (PA) and its various aspects in adolescent students. These included increased levels of PA, support for personal autonomy, intention to engage in physical activity, improvements in body composition, technical performance, fitness level, social support, enjoyment of Physical Education (PE), and positive overall attitudes towards PE classes. Furthermore, based on our findings, TTT has been suggested as a vehicle for fostering social and personal responsibility, as well as promoting enjoyment for adolescents. Therefore, it is crucial that teachers encourage them to be physically and mentally active in order to lead a healthy life.

Our findings are consistent with those of previous reviews and meta-analyses that have highlighted the positive effects of broader educational/multi-component interventions (not exclusively TTT) on the levels of physical activity in adolescents [41,42,43,44,45]. More specifically, García-Hermoso et al., in their meta-analysis, indicated that Physical Education (PE) interventions were positively correlated with improvements in PA and the fundamental motor skills of students, regardless of the frequency or duration of the lessons [43]. Klos et al. further highlighted that PA programs that incorporate group-based learning styles and provide opportunities for voluntary PA are consistently linked to positive PA outcomes [42]. Another systematic review and meta-analysis suggested that implementing multi-component school interventions that incorporate digital components and involve key stakeholders, such as teachers and parents, could potentially help adolescents reach a healthier BMI range [41]. However, a systematic review and meta-analysis suggested that adolescent girls have lower levels of PA than boys and emphasized the value of school-based interventions as a vehicle to improve their PA levels [46]. Nevertheless, our review shows TTT interventions are set apart from the other types of interventions as they specifically target teachers and support them in several ways and are relatively low-cost interventions. More importantly, a major advantage of TTT interventions compared to other interventions is that by supporting/teaching a small population (teachers), they could improve the PA levels of much larger groups (students) [47]. Evidently, TTT interventions could be a valuable tool for education policymakers to improve PA and even the overall quality of education that adolescents receive at school. This is further emphasized in the Comprehensive School Physical Activity Program (CSPAP), which identifies schools (teachers) as playmakers in promoting physical activity and well-being among adolescents [18]. Furthermore, the CSPAP highlights that schools have the capacity to significantly influence the levels of physical activity among adolescents, and it is crucial to utilize their wide reach and resources to offer opportunities and assistance for PA [18].

A major finding of our study was that the majority of the interventions were based on the self-determination theory (SDT), and they positively affected many aspects of PA in adolescent students. A few notable aspects related to STD from our review include autonomy support, social support, enjoyment of PE, and a positive attitude toward PE classes. A potential explanation for this could be that teachers who utilized interventions based on SDT fulfilled the three basic psychological needs of their students: autonomy, competence, and relatedness [48]. Autonomy refers to the need for individuals to feel a sense of ownership over their own behavior. To promote the autonomy of students, TTT interventions could support/guide teachers to structure their lessons based on their students’ needs and give students multiple options/pathways on how to complete tasks while providing them with constructive feedback [49]. Competence, on the other hand, represents the need to achieve desired outcomes and experience a sense of mastery. Lastly, relatedness encompasses the need for individuals to feel connected to others. In order to enhance students’ competence and relatedness, we suggest incorporating active learning strategies such as role-play and team-based learning into TTT interventions [50,51]. This approach could also improve social connectedness (social support) among students and additionally enhance their enjoyment of physical education. By fulfilling their psychological needs, students could be intrinsically motivated to stay physically active. Furthermore, students who fulfilled their psychological needs could enjoy PE more and, as a result, have a more positive attitude towards PE. This means that when designing TTT interventions to improve the PA of students, researchers/policymakers should ensure that teachers better understand their students’ emotions and that students have a positive experience during PE [17]. All of the aforementioned highlight the potential positive effect of utilizing SDT when designing TTT interventions.

Our findings emphasize the potential of TTT interventions as practical and effective methods to increase physical activity levels among adolescents in schools. However, there are several obstacles that could hinder the success of TTT interventions. These obstacles include the limited availability, quality, and cost of teacher training and coaching [35]. Moreover, the sustainability and scalability of these interventions in diverse school environments could also be a challenge. Despite these challenges, TTT interventions have the potential to train teachers in utilizing more efficient and alternative models of education, such as hybrid models [27,29]. This could lead to a more effective improvement in the PA levels of their students, especially during the limited time dedicated to physical education in school [39]. Furthermore, TTT interventions can help teachers in creating positive learning environments and motivating their students to engage in more physical activity both inside and outside of school [28,35]. Therefore, we recommend implementing TTT interventions more frequently in practice while also evaluating and adapting them to meet the specific needs and resources of each school. In conclusion, by adopting TTT interventions, schools can establish a culture of PA that has long-lasting effects on the health and education of future generations.

We propose that schools integrate and incorporate this type of intervention into their regular curriculum and schedule while providing teachers with proper training, support, and feedback. Additionally, policymakers and stakeholders should extend their support and financial resources to these interventions. Moreover, policymakers and stakeholders could develop and implement policies and guidelines that actively promote and facilitate physical activity in schools. Furthermore, we suggest implementing multicenter TTT interventional studies. Such studies would provide valuable insights into the effectiveness of these interventions and the barriers to their implementation across diverse settings and populations, thereby contributing to the refinement of TTT interventions so to enhance their overall impact.

Finally, a study identified six key recommendations by teenagers to improve their PA [4]. These include ‘lower/remove the cost of activities without sacrificing the quality; make physical activity opportunities more locally accessible; improve the standards of existing facilities; make activities more specific to teenagers; give teenagers a choice of activities/increase variety of activity; and provide activities that teenage girls enjoy’ (p. 1) [4]. These recommendations could be implemented in future research on TTT interventions to further optimize them.

### Limitations

Our review, to the best of our knowledge, adds to the existing literature by providing a comprehensive and updated overview of the different types of TTT interventions, outcome measures of the interventions, and their potential effects on adolescents. Despite the valuable insights gained from the present scoping review, it is subject to a number of limitations. First, we included only articles published in English, thus limiting the availability of studies. Second, TTT interventions had different methodologies and were applied to diverse contexts and populations, making comparisons and further analysis difficult. Finally, the quality of the articles was not evaluated, and the conclusions were summarized without any additional analysis.

## 5. Conclusions

In conclusion, we found that Teaching-The-Teachers (TTT) interventions have a positive impact on various outcomes related to PA among adolescent students. These aspects encompass the promotion of personal autonomy, the intention to engage in physical activity (PA), advancements in physical fitness and body composition, social support, satisfaction with Physical Education (PE), and favorable attitudes towards PE. Moreover, these findings indicate that TTT interventions hold significant potential as strategies to promote PA and enhance the physical health of adolescents, particularly within the limited time frame of Physical Education (PE) in schools. Furthermore, implementing TTT interventions could broaden the knowledge and skills of teachers by introducing them to innovative models of PE. Therefore, TTT interventions could contribute to the prevention of non-communicable diseases in the future and improve the overall health of adolescent students. Healthcare and education policymakers could utilize TTT interventions to potentially nurture healthier adolescents and, consequently, societies.

## Figures and Tables

**Table 1:** The PICO methodology for this review.

(P) Population	(I) Intervention	(C) Comparison	(O) Outcome
Adolescents in schools (age 10–18)	Teaching-the-teachers interventions that improve the teachers’ knowledge and/or skills to enhance the physical activity of adolescent students	Adolescent students whose teachers did not receive any ‘teaching-the-teachers’ intervention to improve the physical activity of their students	Improved physical activity of the adolescent students

**Table 2:** Studies investigating the effectiveness of Teaching-the-Teacher (TTT) interventions on improving the physical activity (PA) of adolescent students.

#	Author, Year (Ref.)	Design	Population Characteristics	TTT Interventions	Students’ Interventions *	Outcomes Measures **	Main Findings
1	Engels et al., 2020 [32]	Quasi-experimental	▪ 285 students; ▪ Age 10–16; ▪ Germany.	▪ Teaching in cooperative-games.	▪ 14 cooperative games over 7–14 weeks/lessons, 15 min cooperative games once per week, physical education classes in the intervention group (IG ^2^).	▪ Assessment of Enjoyment in Physical Education questionnaire; ▪ Social relatedness, social interaction, perceived competence, and autonomy scales.	▪ The intervention was positively associated ^+++^ with enjoyment in physical education classes. ▪ Participation in cooperative games ^+^ generated increased enjoyment in physical education classes, strengthened the sense of interpersonal connection among students, and fostered perceptions of competence in the physical education domain. ▪ The impact of cooperative games on enjoyment was partially mediated ^+^ by social relatedness and perceived competence.
2	Fin et al., 2019 [37]	Quasi-experimental	▪ 61 students; ▪ Age 12–14; ▪ Brazil.	▪ Forty (40) h training; ▪ Seminars were conducted to elucidate and deliberate on the techniques for organizing and delivering classes employing an autonomy support style, drawing inspiration from established models.	▪ Duration 8 months; ▪ 55 min in classes on a twice weekly basis for autonomy, interpersonal support program in the IG ^2^.	▪ Autonomy Support Scale (ASS); ▪ Controlling Style Scale (CSS); ▪ Basic Psychological Needs in Physical Education (NPBEF) questionnaire; ▪ Perceived Locus of Causality Questionnaire (PLOCQ); ▪ Physical Activity Enjoyment Scale (PACES).	▪ The intervention was positively associated ^+++^ with higher levels of autonomy support as positive effects from the intervention experienced, as evidenced by their improved scores in areas such as basic psychological needs, intrinsic motivation, self-determined index, and satisfaction with physical education classes (IG ^2^). ▪ Increased indices for autonomy, competence, and relatedness, leading to higher self-determined motivation and satisfaction when participating in physical activity in (IG ^2^).
3	Garcia-Castejon et al., 2021 [27]	Quasi-experimental	▪ 99 students; ▪ Age 12–14; ▪ Spain.	▪ Duration 11 weeks; ▪ Two sessions of 50 min per week; ▪ Training implementation of a hybrid intervention program that combined multisport (initiation to basketball, futsal, and volleyball), objectives and contents for every week, principles and tactical problems, skill-execution task examples, strategies (Teaching Personal Social Responsibility (TPSR) and Teaching Games for Understanding (TGfU) name of the program); ▪ The program incorporated the use of video observation.	▪ Duration 11 weeks; ▪ The students were developed in different tiers of responsibility throughout; ▪ Games and video observation; ▪ Game forms that introduce scenarios that change the nature of the real game.	▪ Mixed methods quantitative and qualitative; ▪ Personal and Social Responsibility Questionnaire (PSRQ); ▪ Motivation questionnaire (PLOC); ▪ Basic Psychological Needs questionnaire (PNSE); ▪ Sport Satisfaction Instrument (SSI) questionnaire; ▪ Questionnaire of intention to be physically active.	▪ The intervention was positively associated ^+++, ##^ with intention to be physically active, as well as in autonomous motivation, the self-determination index, psychological mediators, personal and social responsibility, and enjoyment of physical activity. ▪ The combination of (TPSR) and (TGfU) is outlined as a methodological alternative that can assist in engaging in physical activity.
4	Gil-Arias, A. et al., 2017 [29]	Crossover	▪ 55 students; ▪ Age 15–16; ▪ Spain.	▪ Duration 3 weeks; ▪ A hybrid specialized training program emphasized the sport education model, sparking conversations (Teaching Games for Understanding (TGfU) and Sport Education (SE) name of the program).	▪ Duration 8 weeks; ▪ Sixteen (16) lessons; ▪ Fifty (50) min sessions over the course of one week; ▪ Team sports of volleyball and ultimate frisbee, multiple small-sided competition games.	▪ Perceived Locus of Causality; ▪ Basic Psychological Needs in Exercise scale; ▪ Enjoyment/Boredom in Sport scale; ▪ The Intention To Be Physically Active scale.	▪ The intervention was positively associated ^+++^ with autonomy, competence, and enjoyment of PA. ▪ The variables of autonomous motivation, relatedness, and intention to engage in physical activity showed no significant improvements in one of the groups.
5	Grasten A. & Yi-Piipari S., 2018 [17]	Quasi-experimental	▪ 661 students; ▪ Age 11–13; ▪ Australia.	▪ Training sessions to enhance and expand their physical education instruction methods, which were devised by a group of specialists.	▪ Two (2)-year intervention. The intervention group was provided with physical activity support and task-involving climate support.	▪ Motivation Climate in PE Scale (MCPES); ▪ Health Behavior in School-aged Children Research Protocol; ▪ Accelerometers; ▪ PE Enjoyment scale.	▪ The intervention was not associated ^###^ with any improvements in PA, and there was even a decline in the proportion of children meeting the current MVPA guidelines in both IG ^2^ and control group (CG ^3^), while PE enjoyment remained unchanged in both groups. ▪ Among the (CG ^3^) students, it was only the boys who displayed a higher degree of physical activity than the girls. ▪ According to teachers’ feedback, the program led to a decline in violence and bullying among intervention students, which can be regarded as an extra benefit.
6	Guijarro-Romero et al., 2023 [26]	RCT ^1^	▪ 175 students; ▪ Age 12–16; ▪ Spain.	▪ Advanced training in Self-Determination Theory (STD), with a focus on cultivating autonomy and providing support to fulfill needs in motivation and behavior change techniques; ▪ Guidelines for effectively delivering the lessons in the IG ^2^.	▪ Two (2) Physical Education (PE) lessons per week during the intervention period (IG ^2^ and CG ^3^); ▪ An eight-week program consisting of two teaching units per week, with the goal of fostering healthy physical activity (PA) habits (IG ^2^); ▪ Throughout the entire day, students made use of an activity wristband; ▪ The specific mobile application (Mi Fit) incorporates additional behavior modification strategies, such as educational counseling, physical activity goals, and reminders; ▪ Established a WhatsApp group (IG ^2^) to encourage students’ engagement in physical activities.	▪ Wristbands (accelerometer); ▪ Participants’ body mass, height (BMI) ^4^; ▪ Multi-Dimensional Perceived Autonomy Support Scale for PA (MD-PASS-PA); ▪ Basic Psychological Needs in Exercise Scale (BPNES); ▪ Revised Perceived Locus of Causality scale (PLOC-R); ▪ Behavioral Regulation in Exercise Questionnaire (BREQ-3); ▪ Intention to Partake in Leisure-Time PA questionnaire; ▪ Physician-based Assessment and Counseling for Exercise questionnaire (PACE).	▪ The intervention was positively associated ^+++^ with improvements in cognitive and procedural autonomy support among students (IG ^2^). ▪ The post-intervention to follow-up and pre-intervention to follow-up saw improvements in autonomy and relatedness basic psychological needs, as well as autonomous motivation towards physical activity scores. ▪ The scores for habitual physical activity saw an increase from pre- to post-intervention, and from post-intervention to follow-up.
7	Ha et al., 2020 [39]	RCT ^1^	▪ 667 students; ▪ Age 13–15; ▪ China.	▪ Two (2) trainings, 4 h per workshop; ▪ Training program for a school-based intervention that combined utilization of the fitness dice, adjustment of teacher interpersonal behaviors, and integration of music as a motivating factor during school PE (SELF-FIT name of the program); ▪ Gained expertise in employing the supportive, active, promote students’ emotional well-being, autonomous, fair, enjoyable teaching principles, encompassing a range of strategies to enhance students’ basic needs satisfaction and optimize their engagement in physical activity.	▪ Eight (8) lessons; ▪ Included fitness dice, music.	▪ Accelerometers; ▪ Learning Climate questionnaire; ▪ Perceived Locus of Causality questionnaire; ▪ Flourishing scale; ▪ Basic Needs Satisfaction in Sport scale.	▪ The SELF-FIT intervention was positively associated ^+^ with improvement in students’ physical activity. ▪ IG ^2^ students spent less time in sedentary behaviors and more time in light, moderate, and vigorous activities. ▪ Without the application of the intervention, activity levels within PE and students’ autonomous motivation to PE both declined. ▪ After applying the music component, positive changes in atmosphere and student motivation reported.
8	Hao et al., 2022 [40]	Quasi-experimental	▪ 327 students; ▪ Age 12–14; ▪ China.	▪ Training in in the theory and practice of the well-rounded physical education curriculum model incorporating both theoretical knowledge and practical skills.	▪ Duration 12 weeks (6 weeks long jump lessons, 6 weeks rope skipping lessons); ▪ 3 lessons per week, 40 min; ▪ Physical Education (PE) for the IG ^2^.	▪ Perceived Need Support in PE scale; ▪ Standage Autonomous Motivation in PE scale; ▪ Cause Perception scale; ▪ Behavioral Regulation in Exercise Questionnaire (BREQ-2); ▪ Planned Behavior Theory scale; ▪ Deqing Liang’s physical activity rating scale.	▪ The intervention was positively associated ^+^ with improvements in students’ perception of need support in PE, autonomous motivation in PE, autonomous motivation in leisure time (LT), and participation in extracurricular sports activities for the IG ^2^. ▪ Students’ class motivation can be positively influenced by the supportive behavior of teachers.
9	Lonsdale et al., 2013 [35]	RCT ^1^	▪ 288 students; ▪ Age 13; ▪ Australia.	▪ Training 20 min; ▪ Self-Determination Theory (STD)-based interventions using 3 motivational teaching strategies to develop activity in students.	▪ ‘Free choice’ intervention for warm-up activities and organizing; ▪ Games, dance, netball, and touch rugby.	▪ Wristbands (accelerometer); ▪ Student motivation questionnaires for PE lessons perceived autonomy, competence; ▪ Self-determination Index (SDI).	▪ The intervention was positively associated ^+++^ with students’ perception of autonomy and reduced sedentary behaviors during physical education classes. ▪ The intervention was positively associated ^+++^ with elevated levels of physical activity. ▪ The inclusion of both “providing-choice” and “free-choice” interventions contributed to a reduction in sedentary behavior. ▪ Students’ motivation did not increase during the choice-based interventions.
10	Roth et al., 2019 [38]	RCT ^1^	▪ 3763 students; ▪ Age 12–15; ▪ USA.	▪ Twelve (12) hours training; ▪ Standards-based professional training sessions encompassed didactic instruction, strategies, providing chances to actively participate in PA. Offered curriculum, equipment, and training. Physical education class included time for fitness, skills, classroom management, and play (SPARK name of the program).	▪ Middle school curriculum that contained physical education activities.	▪ PA levels and weekly muscle strengthening (two items adapted from the Youth Risk Behavior Survey); ▪ PE enjoyment (one item adopted from the Amherst Health and Activity Survey); ▪ Attitudes toward PA were assessed using four items regarding respondents’ feelings toward fitness level; ▪ Perceived Barriers to PA scale adapted from the Amherst Health and Activity Study; ▪ Measures of social support for PA were obtained using the family and friend participation subscales of the Sallis Support for Exercise scales.	▪ The intervention was not associated ^++^ with any increase in the number of students engaging in 60 min of exercise per day. ▪ The intervention was negatively associated ^++^ with number of muscle-strengthening exercises. ▪ The intervention was positively associated ^++^ with the enjoyment of physical education and the achievement of passing the Fitness Gram. ▪ There was some indication of a correlation ^++^ between participation in the SPARK intervention and improved fitness level. ▪ There was no indication ^++^ that the intervention enhanced attitudes towards physical activity.
11	Sanchez-Oliva et al., 2017 [28]	RCT ^1^	▪ 836 students; ▪ Age 12–16; ▪ Spain.	▪ The training program is designed to improve teachers’ interpersonal style and empower them with strategies for promoting autonomy, competence, and relatedness need satisfaction, based on Self-Determination Theory (STD).	▪ A didactic unit planned for the “Games and Sport” content block of the educational system.	▪ Basic Psychological Needs Support in Physical Education (CANPB) questionnaire; ▪ Basic Psychological Needs in Exercise Scale (BPNES); ▪ Questionnaire of Motivation in Physical Education (CMEF); ▪ One item to measure students’ intention to practice sport in the following years: “In the following years, I have intention to practice sport”.	▪ The intervention was positively associated ^#^ with students’ (IG ^2^) autonomy support, relatedness support, autonomy satisfaction, autonomous motivation, controlled motivation, and intention to engage in physical activity, in comparison to the (CG ^3^).
12	Schneider, J. et al., 2020 [30]	RCT ^1^	▪ 502 students; ▪ Age 13–15; ▪ Finland.	▪ Duration 2 weeks; ▪ Twelve (12) h training; ▪ Interactive autonomy support teacher-training program, techniques and strategies intended to promote students’ autonomous motivation toward out-of-school physical activities by the Trans-Contextual Model (TCM); ▪ Control group received an alternative training program comprising 4 h of training in one-day workshop for a monitoring system for physical functional capacity for children with special needs (MOVE).	▪ Teachers applied autonomy-supportive techniques in their regular PE classes.	▪ Short form of the International Physical Activity Questionnaire (IPAQ); ▪ International Physical Activity Questionnaire modified to make explicit reference to out-of-school physical activity; ▪ Accelerometers; ▪ Perceived Autonomy Support scale for Exercise Settings; ▪ Perceived Locus of Causality questionnaire; ▪ Sport Motivation scale.	▪ The intervention was not associated ^+^ with any changes in physical activity behavior observed in either group at post-intervention at 1 month. ▪ The perception of support for autonomy had a notable impact on self-motivation in physical education, and self-motivation in physical education had a substantial influence on self-motivation in leisure activities.
13	Sparks, C. et al., 2017 [34]	RCT ^1^	▪ 382 students; ▪ Age 11–15; ▪ Australia.	▪ Three-hour training session on relatedness-supportive teaching practices based on self-determination theory principles, active-learning exercises.	▪ Two or three class periods each lasting 50 min; ▪ Weekly PE intervention to basketball/netball, badminton, and Australian rules football; ▪ The training program resulted in PE teachers adopting instructional behaviors that fostered relatedness.	▪ Perceived Relatedness Support scale; ▪ PE Enjoyment scale; ▪ PE-specific instruments for tripartite efficacy beliefs (self-efficacy, other-efficacy, teacher-focused RISE, and peer-focused RISE); ▪ Relative autonomy index (RAI); ▪ Sport Enjoyment scale; ▪ 20-item Perceived Locus of Causality questionnaire.	▪ The intervention was positively associated ^+++^ with significant improvements in students’ perceptions of relatedness support and enjoyment of Physical Education (PE) compared to the CG ^3^ students. ▪ Students in the IG ^2^ demonstrated increased levels of other-efficacy and peer-focused RISE in contrast to the CG ^3^ students.
14	Springer et al., 2019 [36]	RCT ^1^	▪ 654 students; ▪ Age 11–15; ▪ Uruguay.	▪ Training based in Social Cognitive Theory, Theory of Planned Behavior and socio-ecological models of health behavior. classroom-based; ▪ Curriculum, an afterschool program, activity breaks, and final showcase event (¡Activate Ya! name of the program).	▪ Duration 1 year; ▪ Twelve (12) lessons; ▪ Group training, small group activities, comic book adventure; ▪ The Evento Final, a half-day event that included a showcase of students’ work fun activities and games to promote PA.	▪ A comprehensive past 7-day PA score was created based on responses to eight items from the Physical Activity Questionnaire for Older Children (PAQ-C).	▪ The intervention was positively associated ^+^ with improvements in the past 7-day physical activity (PA) both in general and specifically for girls. ▪ The (IG ^2^) girls reported significantly higher levels of athletic identity, PA competence, friend and teacher PA support at the post-test, as well as increased PA enjoyment at the follow-up.
15	Ten Hoor et al., 2018 [31]	RCT ^1^	▪ 695 students; ▪ Age 11–15; ▪ Netherlands.	▪ Training workshops 15–30 min, two per week; ▪ Physical education and strength exercises; ▪ Specific strength exercises; ▪ Engaging in workshops to enhance their abilities as motivational speakers and equipping them with materials to integrate strength exercises into their lessons; ▪ Received a book with strength exercises and games.	▪ Duration 1 year; ▪ Three lessons of PE per week, 15–30 min per lesson; ▪ Monthly motivational lessons for having been more physically active; ▪ Strength exercises and a motivational intervention to promote after school physical activity (IG ^2^).	▪ Body composition assessed by the deuterium dilution technique; ▪ Anthropometrics were measured using standard procedures (height, weight, Body Mass Index (BMI) was calculated as weight/height; ▪ Daily physical activity and sedentary behavior measured by accelerometry; ▪ Device on students’ lower back with ActiLife software (v6.13.3; https://www.actigraphcor4p.com/support/software/actilife/).	▪ The intervention was positively associated ^#^ with higher fat-free mass and more PA after 1 year that the CG ^3^. More specifically, after 1 year, there was a significant difference in fat mass favoring the IG ^2^ students. ▪ Both groups experienced a decrease in daily physical activity from baseline to post-test, with a smaller decline observed in the IG ^2^ students. ▪ There were no discernible disparities in sedentary behavior or light physical activity among the groups. ▪ The integration of strength exercises and motivational lessons yielded an improvement in body composition and a smaller drop in physical activity level.
16	Tilga et al., 2021 [33]	RCT ^1^	▪ 858 students; ▪ Age 12–15; ▪ Estonia.	▪ 4-week intervention period; ▪ Face-to-face and web-based experimental; ▪ Face-to-face one day in an 8 h workshop; ▪ Study material was delivered to the PE teachers and video lectures.	▪ Autonomy-supportive interventions conducted web-based or face-to-face approach.	▪ Multi-Dimensional Perceived Autonomy Support Scale for Physical Education (MD-PASS-PE); ▪ Multi-dimensional Controlling Coach Behaviors scale adapted to PE; ▪ Basic Psychological Need Satisfaction and Need Frustration scale adapted for PE; ▪ Perceived Locus of Causality questionnaire.	▪ The intervention was positively associated ^+++^ with statistically significant changes in all variables of the study (i.e., a substantial rise in cognitive, organizational, and procedural autonomy-supportive behavior; a boost in psychological need satisfaction for autonomy, competence, and relatedness; and an increase in intrinsic motivation, while observing a marked decline in intimidation, controlling use of grades, and negative conditional regard, as well as a reduction in psychological need frustration for autonomy, competence, and relatedness).

*: The interventions that the teachers who participated in the TTT interventions implemented into their lessons and affected the students. **: How studies measured the effect of the students’ interventions. ^+^ Beta coefficients (β); ^++^ odds ratio; ^+++^ F-value; ^#^ Intraclass correlation coefficient; ^##^ Wilks‘ Alpha; ^###^ standardized root mean square residual (SRMR), the RMSEA, the CFI, and the TLI. Notes: ^1^ (RCT): Randomized controlled trial; ^2^ (IG): intervention group; ^3^ (CG): controlled group; ^4^ (BMI): body mass index.

## Data Availability

No new data were created or analyzed in this study. Data sharing was not applicable in this study.
